# Supportive Interventions Involving Family Carers of Patients With Delirium Superimposed on Dementia in Hospital: A Scoping Review

**DOI:** 10.1111/opn.70016

**Published:** 2025-02-22

**Authors:** Caroline Ashton‐Gough, Jennifer Lynch, Claire Goodman

**Affiliations:** ^1^ University of Hertfordshire Hatfield UK; ^2^ Princess Alexandra Hospital Harlow Essex UK

**Keywords:** carers, delirium, dementia, family caregiving, non‐pharmacological interventions

## Abstract

**Background:**

People with dementia admitted to hospital are at risk of developing delirium. Patients with delirium superimposed on dementia (DSD) have higher mortality rates, longer hospital stays and further cognitive loss. The role of family is often recommended as a resource to inform and support how patients with dementia's needs are understood. This review focuses on ward‐based interventions that enable family carers and health care professionals to work together to improve patient experience and outcomes.

**Aim:**

To review evidence on ward‐based approaches involving family (or their proxies) and staff working together to prevent and manage delirium for patients living with dementia.

**Methods:**

We undertook a scoping review including all types of research. Six electronic databases were searched (CINAHL, MEDLINE (run twice), EMBASE, Cochrane, PsycINFO and PubMed). The search was limited to papers written in English and published from 2009 to 2019. The search was updated in 2023. Papers were independently read by two researchers. Findings were presented through narrative synthesis (Prospero CRD42019130369).

**Results:**

Fifteen papers were included. Studies focused on educational and system change to improve the care of people with DSD. Family involvement ranged from enabling baseline assessment of delirium, commenting on different resources and measures designed to support their involvement in care or simulate their presence. The evidence of effectiveness was varied. Interventions to support personalised care and give family carers and staff confidence were positively evaluated in some studies but not all included both family carers and staff. Benefits to patients over time were less clearly demonstrated.

**Conclusion:**

This review identified the potential of family to mitigate the risk of delirium and improve patient outcomes. Further research is needed to understand how system and practitioner changes to enable family involvement in the support of people with DSD benefit patients in the short and long term.

**Relevance to Clinical Practice:**

The review findings provide evidence for clinical practice when selecting existing interventions and approaches involving family in supporting patients with DSD.

**Patient or Public Contribution:**

Not required as this was a review, not an original piece of research.

**Summary**

- What does this research add to existing knowledge in gerontology?
  ○ This scoping review shows there are few supportive interventions involving family carers for patients with dementia in hospital at risk of or experiencing delirium.
  ○ International studies of supportive interventions for patients with dementia and superimposed delirium, which have focused on family involvement, are clinically heterogeneous, differ in design, implementation and follow‐up times.
  ○ International studies of supportive interventions involving family carers for patients with dementia and superimposed delirium often focus on staff workload and families' satisfaction with care rather than patient‐centred outcomes.
- What are the implications of this new knowledge for nursing care for and with older adults?
  ○ There is a consensus among the studies included in this scoping review that involving family/carers of patients with dementia at risk of/with delirium should improve the patient experience.
  ○ There is limited evidence on how professionals should work with family of patients with dementia at risk of/with delirium and others to improve delirium recognition and reduce its impact.
- How could the findings be used to influence practice, education, research, and policy?
  ○ The findings of this study can be used to raise awareness in clinical practice about working with family members in supporting patients with delirium superimposed on dementia.
  ○ The findings of this study demonstrate the need for further guidance on developing effective interventions and clinical guidelines for involving family members in supporting patients with delirium superimposed on dementia.

## 1 Introduction

In England, people living with dementia are disproportionately admitted to hospital and are more likely to experience longer stays (Briggs et al. 2016; Crowther et al. 2017; Mukadam and Sampson 2011; Timmons et al. 2015). Patients in hospital living with dementia are at a high risk of developing delirium. International research reports on rates of delirium superimposed on dementia (DSD) in older hospitalised patients as ranging from 22% to 31% (Fick et al. 2002; Avelino‐Silva 2017; Morandi et al. 2017).

Dementia and delirium are distinct but interrelated conditions. Delirium is an ‘acute disturbance of consciousness and cognition that develops over a short course with fluctuating symptoms’ that can affect all clinical conditions (O'Hanlon et al. 2014; Roden and Simmons 2014). For many patients, incident delirium can be avoided if suitable recognition and avoidance strategies are in place (Anderson 2005; Fong et al. 2009; Fong and Inouye 2022). The occurrence of delirium in a person with dementia, DSD is often unrecognised in acute settings (Morandi et al. 2017). DSD has been independently associated with adverse outcomes, including prolonged hospital stay, functional and cognitive decline and mortality (Grossi et al. 2020). The experience of the pandemic has illustrated how important it is for nurses to address delirium in patients with dementia and acute infections (Bianchetti et al. 2022; Wang et al. 2020).

Research on family involvement in the management of delirium and/or dementia has argued that participation in care can increase patient and family satisfaction, as well as improve patient outcomes (Davies et al. 2014; Davis et al. 2019; Fawcett 2011; Johnson 2000; McKenzie and Joy 2020; Young et al. 2010). Family caregivers, however, often feel their knowledge of their family member and how dementia affects them is undervalued and unrecognised by ward staff (Julian et al. 2022; Schmitt et al. 2017). The additional experience of caring for someone with delirium can increase caregivers' distress and sense of uncertainty (Shrestha and Fick 2020). A review into the interrelationship between delirium and dementia and delirium prevention interventions suggested family carers should be actively involved (Fong and Inouye 2022).

Research on family carers of patients with dementia and delirium in hospital settings is limited (Halloway 2014; McKenzie and Joy 2020), and literature on dementia friendly hospital settings in England often focuses on environmental issues (Handley et al. 2019). This paper reports on supportive interventions involving family carers on the health outcomes of older adults with dementia who are at risk of or experience delirium during hospital admission.

We sought to address the following questions:

- What kind of organisational, educational and peer support can deliver an improved nurse–family carer relationship for delirium prevention for hospital patients with dementia?
- What is the evidence that approaches that involve patients with DSD, nurses and their relatives/supporters lead to improved outcomes for the patient and experience for the carer?

## 2 Methods

### 2.1 Study Design

A scoping review is an appropriate method for identifying the types of available evidence, synthesise existing research findings and investigate gaps in the research (Peters et al. 2020). It is a useful approach for studying emerging evidence when an overview of existing research is required before more precise questions can be addressed by a systematic review (Munn et al. 2018). We chose to undertake a scoping review to map existing evidence on this under‐investigated topic. This scoping review used an adaptation of the PCC (population, concept and context) framework suggested by Joanna Briggs Institute (Peters et al. 2021) to produce specific research questions (Table 1). We followed the five methodological stages for undertaking a scoping review suggested by Arksey and O'Malley (2005): (1) identification of the research question; (2) identification of relevant studies; (3) study selection; (4) charting the data; and (5) collating, summarising and disseminating the results. The review is structured in accordance with the Preferred Reporting items for Systematic Reviews and Meta‐Analyses (PRISMA‐ScR) checklist, which provided us with 20 items that supported the development and reporting of this scoping review (Peters et al. 2021; Tricco et al. 2018). The protocol was registered with the PROSPERO database (CRD42019130369).

**TABLE 1:** An illustration of the PCC for this review.

Population	Concept	Context
Inpatients with dementia and delirium	Supportive interventions involving family carers	Hospital
Family		
Older adults		

### 2.2 Population

The sources report on older people or adults aged 65 years and above, with dementia and delirium and their families, spouses or significant others (hereafter, referred to as family carers or caregivers).

### 2.3 Concept

The review was interested in supportive interventions involving family carers when providing care for patients with DSD.

### 2.4 Context

The review focused specifically on hospitals providing inpatient care.

### 2.5 Search Strategy

Our search strategy for this scoping review drew on the approach described by Peters et al. (2021), to ensure a comprehensive conceptual map of the topic within the constraints of time and resources. We implemented no limits on methods or methodology in mapping the relevant literature on our topic.

First, we performed an open limited search in the Google Scholar search engine. The aim of this search was to get an overview of the topic and to get acquainted with the volume of literature on the topic, relevant index terms and keywords.

Then, we conducted a systematic scoping search in the databases of CINAHL, PsycINFO, MEDLINE, PUBMED, Embase and Cochrane. In this phase, we looked for the database index words, such as headings and MeSH terms. The search was limited to papers written in English and published between January 2009 and December 2019 and updated in 2023.

The first author reviewed the identified sources and studied their bibliographies to ensure all relevant studies were included in the scoping review. All sources were then reviewed and verified by the second author. The search was performed with the assistance of a clinical librarian. In Table 2, we present an example of the building blocks in the search from the CINAHL database. The full search protocol is reproduced in Appendix 1.

**TABLE 2:** Example of the building blocks used in one database search.

Building blocks in the CINAHL search				
OR	AND			
	exp “COGNITION DISORDERS”[MH] exp. “DEMENTIA”[MH] cognitive ADJ3 impairment cognitive impairment* cognitive disorder* cognitive ADJ3 disorder* dementia	“exp DELIRIUM”[MH] delirium	exp “HOSPITALS, DISTRICT” exp. “HEALTH FACILITY ENVIRONMENT” hospital* ward* NOT exp. “COMMUNITY HEALTH SERVICES” communit*	exp “NURSING”[MH] nurs*

### 2.6 Eligibility Criteria and Study Selection Process

A preliminary review of forward citations from papers published in the 1990s discussing DSD found a focus on symptoms, incidence and assessment rather than care based interventions involving family and nursing staff. We were therefore confident that 10 years of literature would identify the most relevant research for this review. We used the following inclusion criteria: (1) original research, including both quantitative and qualitative research; (2) research published between 2009 and 2019; (3) research related to patients with dementia and delirium; (4) research in hospital settings; (5) research that focused on supportive interventions involving family carers. We included studies reporting on all intervention and observational designs that focused on family or their proxies (e.g., unpaid volunteers) being involved in the care of patients with DSD. Inclusion criteria were studies involving the following:

- Hospital inpatients with dementia (all types and stages) or cognitive impairment;
- Inpatients with delirium superimposed on dementia/cognitive impairment;
- Patients with dementia identified as at risk of developing delirium;
- Unpaid volunteers who could represent the needs of the patient;
- Nurses caring for people with dementia and delirium;
- The multidisciplinary team working with patients with dementia and superimposed delirium.

Studies were excluded if

1. conducted with older people with dementia and delirium in a long term or community setting;
2. they did not include people with dementia with or at risk of delirium;
3. we could not distinguish information about family or family proxy activities from that of the health care professionals;
4. published before 2000.

After pilot testing, the eligibility criteria by the three authors using some sample studies, the first author screened titles and abstracts applying selection criteria to potentially relevant papers. If it was unclear if they should be included these studies were reviewed by the other authors.

### 2.7 Data Extraction

One researcher (CAG) independently extracted the data using a structured data extraction template. The process was later verified by the other two authors. Duplicates were identified and excluded. We used an excel spreadsheet to describe the outline of the studies, publication year, aims/research questions, study design, study population, research methodology, type of intervention, participants and outcomes. Reference lists of included studies were reviewed to identify any relevant papers not captured through electronic searches. Study characteristics were tabulated and study outcomes were extracted, assessed and categorised using elements from the JBI tools for data extraction (Table 3).

**TABLE 3:** Study characteristics.

Author, year and location	Study design and setting	Number of participants, age and condition	Type of family carer intervention	Comparison/control and duration of data collection	How dementia and delirium was assessed	Outcomes measured
Ayton et al. (2020) Australia	Mixed methods. Acute and sub‐acute metropolitan hospital (Pre‐implementation study)	Nurses (*N* = 73) (returning surveys) patient/caregiver dyad interviews (*N* = 4) Key stakeholder interviews (consultant, OT, volunteer coordinator, etc.) (*N* = 7) condition—dementia/delirium	1:1 companionship for patients with dementia and/or delirium provided by volunteers	Not reported	Not reported	Acceptability of the programme for key stakeholders—patients, caregivers, nurses, hospital staff and volunteers
Bateman et al. (2016) Australia	Quasi‐experimental; pre‐post design. Acute rural hospital	Patients Aged > 65 years |(or > 50 years for Aboriginal persons) (*N* = 64) dementia/delirium Diagnosis, known risk factors for Delirium or SMMSE < 25/30	Volunteer training programme; including completion of personal profile with the patient or family carer	Data collected at baseline and at 8 months	A diagnosis of dementia OR A diagnosis of delirium OR mini mental state Examination (SMMSE) score of < 25/30 OR one or more risk or precipitating factors for delirium	Patient outcomes: Use of antipsychotics/psychotropic medications; use of analgesia, use of antidepressants, length of stay, number of falls, incidence of delirium staff/volunteer outcomes staff: attitude to PCC Volunteers: knowledge and confidence. Attitude to PCC
Blair et al. (2018) Australia	Non‐randomised controlled trial. Seven acute rural Hospitals located in Southern New South Wales local health district (SNSWLHD).	Patients (*N* = 270); family members (*N* = 80); staff (survey) (*N* = 119); staff (focus groups) (*N* = 46); volunteers (*N* = 44). Patients had dementia or delirium Condition—dementia and/or delirium	Volunteer training programme; Completed a Personal Profile with the family carer Family interviews/surveys Focus groups	Control group of historical patients admitted 12 months prior to programme. Beginning of Project (data recorded over 8 months)	Not reported	Increased safety and quality of care. Increased patient well‐being. Reduction in family care burden. Reduction in nursing care burden
Boltz et al. (2015) USA	Comparative repeated measures study. Five medical units in two hospitals in Northeast USA	Patients (older adults aged 65 or over); English speaking/reading; a positive mini‐cog; Intervention group *N* = 44; nonintervention group *n* = 42; Family caregiver *N* = 86 Dyads	A function focused model of care intervention including four components, (1) environmental and policy assessment, (2) staff education, (3) individualised goals, (4) motivation of nursing staff/patients	One intervention unit in each hospital, three control units Over 18 months	A positive mini‐cog assessment	Patient outcomes: ADL performance; walking performance; gait and balance; delirium severity; hospital discharge outcomes. Family caregiver measures: preparedness for care‐giving; anxiety and depression; strain; mutuality between caregiver and care receiver
Collier et al. (2020) Australia	Qualitative, using video reflexive ethnography. Specialist older people and evaluation management unit in a sub‐acute hospital	Patients (*n* = 3); family members (*n* = 5); managerial staff members (*n* = 2); clinical staff members (*n* = 35); and non‐clinical staff members (*n* = 5). Age—not reported Condition—dementia and delirium	Observation of patients, family and staff members.	21 months	Not reported	Team commitment to high quality fundamentals of care. Teamwork. Dementia‐friendly environment. Aptitude to working with people with dementia
Fick et al. (2011) USA	Prospective cohort pilot study testing feasibility of one component of multicomponent intervention. One adult medical surgical unit in an acute care hospital	Patients *n* = 15 Patients/family *N* = 3 Aged over 65, mean age 83. Dementia diagnosis	Computerised decision support screen (for delirium assessment and management) component of multicomponent intervention for early nurse detection of delirium superimposed on dementia. Study followed consecutively admitted patients and their caregivers for duration of admission	Study carried out over 14‐week period Patients and family carers followed for duration of hospital stay	Aged 65 and over, met criteria for dementia using MBDRS screen. Family caregiver was interviewed using two instruments Modified Blessed Dementia Rating scale and Clinical Dementia Rating Scale. Daily assessment of delirium using a structured interview consisting of MMSE, Observation and the Confusion Assessment Method (CAM)	Nurse adherence to and usability of electronic medical record documentation. Narrative feedback on screens. Patient and family satisfaction surveys post discharge
Goldberg et al. (2013) England	Randomised controlled trial. Large acute general hospital in the UK Medical/mental health unit (28beds)	*N* = 600 patients (310 from specialist unit, 290 from standard care) *n* = 250 sets patient notes Age over 65 years. Patients with dementia and/or delirium	Family carers were recruited, if available and willing to act as an informant. Proactive and inclusive approach to family carers	Randomised to either usual care ward or specialist medical and mental health unit. Recruitment over 18 months Follow‐up completed at Month 21.	Not recorded. Patients included identified as ‘confused’ on admission (mitigating overlap in delirium and dementia) but not requiring other specialist care	Number of days spent at home (or in the same care home) in the 90 days after randomisation. Quality of life. Behavioural and psychological symptoms. Physical disability. Cognitive impairment (MMSE); Inpatient falls. Mortality. Patient experience. Length of stay. Carer strain. Carer wellbeing. Carers' satisfaction
Kang et al. (2017) South Korea	Mixed‐methods sequential explanatory, pre‐post design. One regional general hospital	RN's *N* = 40 recruited for education programme. Purposive sample of RN's *N* = 12 Family Caregivers *N* = 6 No patients recruited	Individual interviews. Delirium brochures given to family caregivers as part of nurse education programme	Conducted between July 2013 and March 2014. RNs completed surveys at baseline and 3 months after the educational programme Qualitative interviews, 2 months after the programme	Not reported	Impact of education programme. Nurses' knowledge of cognitive impairment. Attitudes to older adults. Nurse initiated efforts to involve family caregivers
Martinez et al. (2012) Chile	Single‐blind randomised controlled trial. Internal medicine ward	Patients *N* = 287 Age‐ > 70 years Condition—Previous history of cognitive impairment	Non‐pharmacological intervention delivered to and by family members, including education on delirium; avoidance of sensory deprivation; presence of familiar objects; provision of clock and calendar in the room; reorientation of patient by family; extended visitation times	Standard care (*N* = 143). Allocated to multicomponent intervention (*N* = 144). Patients recruited over 8 months with follow‐up until last hospital discharge	All patients at risk of delirium on basis of 1 risk factor < 24 on mini‐mental state examination	Presence of delirium assessed by CAM. Incidence of falls during hospital stay and complications derived from them
Mailhot et al. (2020) USA	Validation study. Urban academic emergency department (ED).	Dyads of ED patients aged 70 and older and their family caregivers (*N* = 108 dyads). Condition‐with/without dementia	Family caregiver self‐administered the family confusion assessment method (FAM‐CAM) independently	For concurrent validity, performance of the FAM‐CAM was compared to the reference‐standard confusion assessment method (CAM). For predictive validity, clinical outcomes over 6‐months were compared by FAM‐CAM status (positive/negative)	The Informant Questionnaire on Cognitive Decline in the Elderly (IQCODE) used for caregiver assessment of the patient's level of cognitive functioning	Performance characteristics of the FAM‐CAM method. Clinical outcomes 6 months post enrolment by FAM‐CAM status: ED visits, hospitalisation and mortality
Paulson et al. (2016), USA	Education initiative implementation within large multisite intervention study. 24‐bedded inpatient medicine and Acute Care of the Elderly unit (ACE) at a large medical centre	Nurses (*N* = 32) and ancillary staff (*N* = 14) given brochure. Nurses (*N* = 7) completed feedback survey. No patients recruited	Delirium education brochure for family caregivers. Shared with nurses to be used with family caregivers	Implemented over 16 months	Patient data not included	Nurse feedback on use and usefulness of a delirium education brochure for family carers and staff
Teodorczuk et al. (2014) UK	Implementation and evaluation study. One regional hospital trust	Health care professionals (*N* = 48) representing 12 different professional groups. No patients recruited	Training programme underpinned by learning from patients and family carers	Two‐day interprofessional dementia and delirium education programme. Course implemented three times over 18 month period	Patient data not recorded	Participants' confidence in certain acts of professionalism related to care of confused older patients. Changes in attitudes and knowledge
Waszynski et al. (2018) USA	Single‐site randomised controlled trial. Mixed factorial design. Acute care, level 1 trauma centre in an inner‐city state	Hospitalised patients with dementia experiencing delirium (*N* = 126). Family members (*N* = 56) participated in the production of family video messages	Family members recruited to produce video message	Family video intervention compared to nature video intervention and usual care. Intervention × 4 time points, conducted over 9 months	Hyperactive or mixed delirium evidenced by positive score on CAM, and a score of > 0 Richmond Agitation Sedation Scale (RASS), Dementia assessment not recorded	Medication administration for decreasing agitation. Participant agitation (ABS score)
Wong Shee et al. (2014) Australia	Qualitative design. 30 bed Inpatient rehabilitation unit, large regional health service	Patients (*N* = 30) Carers (*N* = 3) Volunteers (*N* = 10) Staff (*N* = 6)	Volunteer diversional therapy programme	Study conducted over 6 months.	Cognitive impairment, defined as a Mini Mental State Examination (MMSE) score < 25 25 or a diagnosis of dementia, and had exhibited behavioural disturbance (e.g., agitation or wandering)	Staff, volunteers and patients/carers perceptions (acceptability and feasibility) of intervention
Yevchak et al. (2017) USA	Qualitative, exploratory, descriptive study within cluster randomised trial. Across three regional and academic medical centre sites	Patients (*N* = 803) 51.4% of delirium rounds included research staff and unit champion Number of family carers not reported Patients with dementia and/or delirium	Weekly delirium rounds led by advanced practitioner nurse (*N* = 750). Staff were encouraged to talk with patients and family members to learn more about hobbies, interests and occupation	Analysis of delirium rounds over 38 months	Not reported	Instances of person‐centred care during delirium rounds

### 2.8 Quality Appraisal

Critical appraisal is not necessarily required for a scoping review (Arksey and O'Malley 2005), and Joanna Briggs Institute (JBI) guidelines specify that scoping reviews are designed to map the breadth and extent of evidence without necessarily evaluating the quality of included studies. The authors, however, believed that assessing quality provided additional insight into the robustness of the evidence to enhance the utility of the findings for practitioners and future work. Articles that met the inclusion criteria were assessed using the JBI standardised critical appraisal checklists (Lockwood et al. 2015; Tufanaru et al. 2020) (Appendix 3, Tables A2 and A3). Two authors reviewed all the papers.

### 2.9 Data Synthesis

Studies were organised initially by descriptive category, for example, participant characteristics (staff, family and patients) and findings synthesised to identify learning on approach and effectiveness. Qualitative data were organised under descriptive categories, discussed and then coded into categories that captured the assumptions of the intervention and the experiences of participants. Outcomes were listed and tabulated. The key findings are presented in a table with a narrative summary to provide an overview of the available evidence base on the review questions (Appendix 2, Table A1).

## 3 Results

### 3.1 Literature Search and Article Selection

**FIGURE 1:**
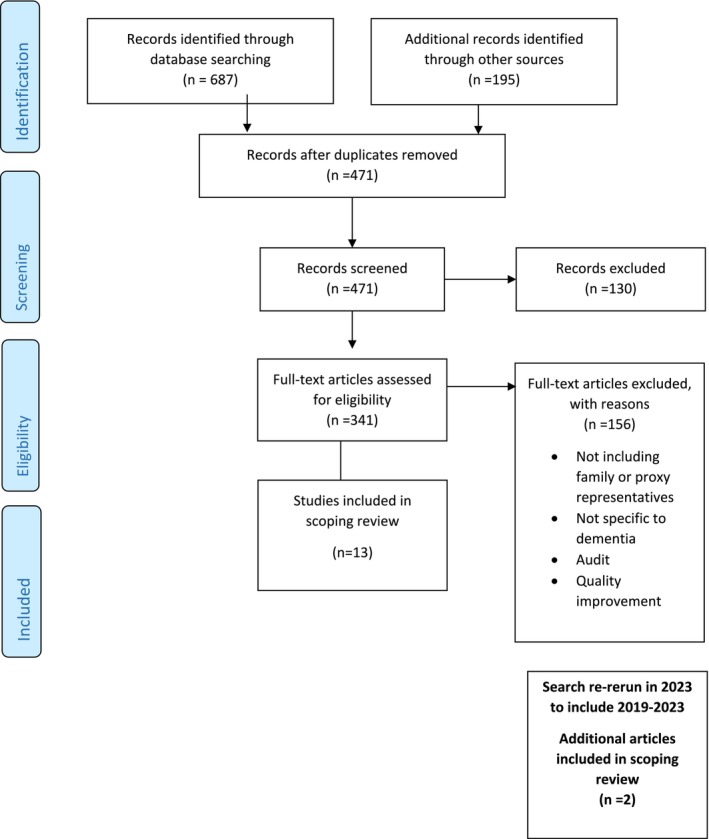
PRISMA flow chart—search 2009–2019/updated 2023.

As shown in Figure 1, the initial literature search yielded 2976 articles, leaving 1486 after duplicates were removed. After review of titles and abstracts, 198 articles remained. Full‐text review left 13 articles meeting inclusion/exclusion criteria. The original search completed in 2020 was re‐run in 2023, yielding two additional papers for inclusion. Figure 1. Therefore, a total of 15 articles were included in this review.

### 3.2 Study Quality

Of the 15 studies included in our review, eight were classified high quality (higher than 70%) (Ayton et al. 2020; Bateman et al. 2016; Blair et al. 2018; Collier et al. 2020; Goldberg et al. 2013; Martinez et al. 2012; Teodorczuk et al. 2014; Waszynski et al. 2018) and seven were of medium quality (see Tables A2 and A3 in Appendix 3) (Boltz et al. 2015; Fick et al. 2011; Kang et al. 2017; Mailhot et al. 2020; Paulson et al. 2016; Wong Shee et al. 2014; Yevchak et al. 2017). The three randomised controlled trials appraised using the JBI tool for RCTs were all classified high quality, while there was more variability overall among the studies of other design. Of the 12 studies appraised using the JBI tool for qualitative research, all but two (Kang et al. 2017; Paulson et al. 2016) clearly demonstrated congruity between research methodology and research question, methods and philosophical perspective. Studies were less successful at locating the researcher theoretically or addressing researcher influence on the research.

### 3.3 Study Characteristics

Of the 15 studies, six studies were conducted in the United States of America (USA) (Boltz et al. 2015; Fick et al. 2011; Mailhot et al. 2020; Paulson et al. 2016; Waszynski et al. 2018; Yevchak et al. 2017), five in Australia (Ayton et al. 2020; Bateman et al. 2016; Blair et al. 2018; Collier et al. 2020; Wong Shee et al. 2014), two in the United Kingdom (UK) (Goldberg et al. 2013; Teodorczuk et al. 2014), one in Chile (Martinez et al. 2012) and one in South Korea (Kang et al. 2017). Settings varied from rural to metropolitan hospitals, with most studies conducted in older people's wards including rehabilitation, specialist medical/mental health, and surgical wards. Three studies were randomised controlled trials (Goldberg et al. 2013; Martinez et al. 2012; Waszynski et al. 2018). The remaining studies included qualitative methods (Collier et al. 2020; Mailhot et al. 2020; Paulson et al. 2016; Teodorczuk et al. 2014; Wong Shee et al. 2014; Yevchak et al. 2017), mixed methods (Ayton et al. 2020; Blair et al. 2018; Kang et al. 2017), prospective cohort (Fick et al. 2011), quasi‐experimental (Bateman et al. 2016) and comparative (Boltz et al. 2015).

Duration of data collection varied and was often reported as a period of observation. Ward‐based observations ranged from 2 days to 18 months. The characteristics of the 15 studies included in this review are outlined in Table 3.

The definition of ‘older adult’ ranged from 50 years and above. Not all studies reported participants' ages. Bateman et al. (2016) specified participants were aged older than 65 years or older than 50 years for Australian Aboriginal participants. Dementia was diagnosed using a range of validated measures, including The Modified Blessed Dementia Rating Scale (MBDRS) (Fick et al. 2011), AD8 score (Boltz et al. 2015) and Mini‐Mental State Examination (MMSE) (Bateman et al. 2016). One RCT (Martinez et al. 2012) reported a low incidence of dementia 2%–6% of the included patients within both groups.

### 3.4 Summary of Included Studies

Supportive interventions ranged from those involving family in keeping the patient active and oriented to those that focused on staff knowledge including identification of people at risk, raising awareness of how family members could inform care, the admission process and ward environment (see Table A1 in Appendix 2).

Five studies emphasised staff education and training to enable family engagement (Boltz et al. 2015; Kang et al. 2017; Mailhot et al. 2020; Paulson et al. 2016; Teodorczuk et al. 2014). One RCT used video recording to simulate the family member's presence as a non‐pharmacological intervention to reduce agitation in persons with dementia (Waszynski et al. 2018). Other studies included educational packages for nursing staff (Fick et al. 2011; Kang et al. 2017; Paulson et al. 2016; Teodorczuk et al. 2014; Blair et al. 2018; Boltz et al. 2015). In four studies, volunteers were recruited as a proxy for a family member and where appropriate worked with a family carer (Ayton et al. 2020; Bateman et al. 2016; Blair et al. 2018; Wong Shee et al. 2014). They were supported to engage in conversations to help orient the person, assist with feeding, hydration, mobilisation, use of visual and/or hearing aids, meaningful activities and general encouragement for patients. One feasibility study tested a family‐centred, function‐focused care intervention (Fam‐FFC); this involved staff education, environmental adaptations and a nurse to support family engagement in activities supporting nutrition, physical activity and cognitive stimulation (Boltz et al. 2015).

Some of the included studies involved family members in as much as they were invited to provide feedback on the intervention. One RCT (Goldberg et al. 2013) tested a specialist medical and mental health unit for people living with dementia and included family experiences of care in the outcome measures. They compared the outcomes (patient health status outcomes, carer strain, carer's psychological well‐being, length of stay, agitation, days spent at home post discharge) of patients admitted to a specialist unit delivering best practice care for people with dementia/delirium compared to standard care (Goldberg et al. 2013). Staff were trained in recognition and management of delirium and dementia and the delivery of person‐centred dementia care and facilitated a proactive and inclusive approach to family carers. Results showed outcomes were no different between groups 90 days after randomisation. This was explained as due to the severity of the patients' dementia, many of whom were nearing end of life (Goldberg et al. 2013). There were, however, improvements in the patient experience and family carer satisfaction. Family carers of patients randomised to the specialist unit were significantly more satisfied with overall care, nutrition, dignity and respect, how the needs of confused patients were met and discharge arrangements.

Two studies focused on delirium assessment, the implementation of a delirium round across three hospital settings and a computerised decision support tool to assess DSD (Yevchak et al. 2017; Fick et al. 2011).

Family or their proxies were often cited as key informants about their relative for the development of training resources, including videos and brochures and details about the patient (Bateman et al. 2016; Blair et al. 2018; Kang et al. 2017; Mailhot et al. 2020; Paulson et al. 2016; Teodorczuk et al. 2014).

Across the 15 studies, patient‐focused outcome measures included length of stay (Bateman et al. 2016; Boltz et al. 2015; Goldberg et al. 2013; Martinez et al. 2012); patient behaviour or evidence of distress (Blair et al. 2018; Waszynski et al. 2018); falls and functional abilities (Boltz et al. 2015; Martinez et al. 2012); increased use of analgesia (Bateman et al. 2016; Blair et al. 2018); mortality (Goldberg et al. 2013); incidence of delirium (Bateman et al. 2016); reduced severity of delirium; and improved activities of daily living performance (ADL's) (Boltz et al. 2015). Family carers' satisfaction with the care their relative received was the outcome measure for six studies (Boltz et al. 2015; Collier et al. 2020; Fick et al. 2011; Blair et al. 2018; Goldberg et al. 2013; Kang et al. 2017). Reduction in family caregiver burden was reported in Blair et al. (2018). Staff‐focused measures considered the feasibility of the intervention (Ayton et al. 2020; Wong Shee et al. 2014) and impact on staff workload (Bateman et al. 2016; Blair et al. 2018).

## 4 Findings

We synthesised our study findings to identify prominent or recurring themes. For the qualitative data, we drew on both quotes and text in the findings section of the studies. These could be summarised under four recurring themes: (1) organisational, (2), educational (3) peer support and (4) interventions directly involving families.

### 4.1 Organisational

How the organisation and culture of the ward affected staff engagement with patients with DSD and their families was identified in three studies. One Australian single‐site ethnographic study focused on the fundamentals of care to deliver high‐quality care for people with dementia and delirium. Fundamentals of care were defined as optimising sleep, hydration and nutrition, vision, hearing and cognitive and physical activities combined with nursing staff knowing the patient sufficiently well to anticipate and communicate their needs. This was supplemented with role modelling by the unit manager (Collier et al. 2020). Over an 18‐month period, using video reflexive ethnography in a specialist older people evaluation unit they explored how care based on these principles was provided. Qualitative findings suggested that creating a special space, involving nurses committed to the care of older people living with dementia who listened to family members and a commitment to teamwork were important. The authors argued it was the collective values and philosophy of the team (rather than an individual's actions) and the trusting nurse, patient and family relationships that were central to placing high value on and enacting high‐quality fundamentals of care. The transferability of the findings should be considered with caution as only 3 patients and 5 family members consented to participate and findings reflected staff (*n* = 40) accounts.

Other organisational findings considered the feasibility of the intervention for staff to implement (Ayton et al. 2020; Wong Shee et al. 2014) and impact on staff workload (Bateman et al. 2016; Blair et al. 2018). They found that it was possible to incorporate family members into the organisation of care and was perceived to reduce staff workload in some studies.

### 4.2 Educational

The studies that included a focus on the education of family caregivers intended to improve nursing staff and families' confidence in family members taking on a caregiver role on the ward (Bateman et al. 2016; Boltz et al. 2015; Fick et al. 2011; Kang et al. 2017; Martinez et al. 2012; Paulson et al. 2016; Teodorczuk et al. 2014; Wong Shee et al. 2014; Yevchak et al. 2017). For several studies educational support was represented as part of the wider multidisciplinary team's work and responsibilities (Boltz et al. 2015; Goldberg et al. 2013; Paulson et al. 2016).

Educational interventions consistently increased staff knowledge of the patient and preparedness for caring and staff and family confidence in managing patients' symptoms. However, there was limited evidence about efficacy and improvement in delirium recognition and care for the patients themselves. One study (Teodorczuk et al. 2014) addressed eight specific learning needs: (1) clinical leadership, (2) negative attitude, (3) understanding how frightened the patient is in hospital, (4) carer partnerships, (5) person‐centred care, (6) communication, (7) recognition of cognitive impairment and (8) staff‐specific learning needs. They found a positive attitude change in this patient group, although actual practice change was not evaluated. The study that offered a delirium admission brochure for family members designed to aid in the detection and early prevention of delirium during hospitalisation assessed the post discharge satisfaction of some family and patients (Paulson et al. 2016). It reported family members were interested in using the brochure. However, its implementation was piecemeal and the evaluation did not include family members focusing on staff perceptions of usefulness.

An educational intervention as part of a larger study included nurse training, a decision support tool, a delirium champion and weekly review ‘round’ led by a specialist nurse. To reduce and manage the risk of delirium of people with dementia nursing staff were encouraged to talk to patients and family members to learn more about hobbies, interests and occupation (Yevchak et al. 2017). The qualitative findings suggested it was possible to apply the principles of person‐centred care for patients with DSD and their carers but recognised this did not always fit with nurses' other priorities. The authors stressed the importance of knowing the patient's baseline characteristics and the implementation of rounds was judged successful. Whether it affected how staff and family members worked together was not described.

### 4.3 Peer Support

Not all patients with dementia have family or friends to support them. More than one study used volunteers, as family proxies, to support the care of patients with dementia and delirium. In a 63‐bed acute rural hospital in Australia, volunteers were trained to provide person‐centred care for patients with dementia/delirium (Bateman et al. 2016). Volunteers completed a 4‐day training programme, and all volunteers stayed in the programme for 6 months. Regular meetings were held with the nurse manager to monitor progress and address any issues. The observational study identified a significant reduction over time in length of stay for patients, and an increase in the use of analgesic medications.

One study in seven acute rural Australian hospitals explored how volunteers contributed to the quality of care for older patients and reduced staff and family care burden (Blair et al. 2018). The intervention included recruiting and training volunteers to provide one‐to‐one practical assistance (e.g., assisting with eating, drinking, walking and use of visual aids, therapeutic activities and emotional support). Family/carers were not involved in the development or in receipt of the training. Family interviews revealed that 96% (*n* = 77) reported positive feedback. Similarly, 97% of staff felt the programme supported nursing care. Key challenges to the programme included initial role delineation, staff/volunteer trust and sustainability. A smaller Australian study in a 30‐bed rehabilitation unit (Wong Shee et al. 2014) explored the acceptability and feasibility of a diversional volunteer programme with patients with cognitive impairment including dementia. Outcomes overall identified perceived benefits for quality of care.

### 4.4 Interventions Directly Involving Family

Waszynski et al. (2018) explored the use of simulated family presence (SFP) on the agitation level of hospitalised, delirious, acutely agitated patients. Family members were asked to pre‐record a video message. Participants included in the study were those experiencing hyperactive or mixed delirium and receiving continuous observation (*n* = 126) with 111 completing the study, of whom 67 had dementia. Participants were randomised to either view a 1‐minute family video message, a 1‐minute nature video or usual care. The Agitated Behaviour Scale measured agitation prior to, during, immediately following and 30 min following the intervention. There were small but statistically significant findings to support the use of family video messaging to decrease agitation and evidence that a nature video was preferable to no intervention. The authors concluded that further work was justified to see if the positive results could be sustained.

One prospective study tested patient family‐carer dyads from admission to the emergency department for delirium and included people with dementia (Mailhot et al. 2020). Patients' outcomes were followed up via health records for 6 months. The FAM‐CAM (family confusion assessment method to identify delirium) intervention was intended to educate family members in recognising delirium. It was sensitive to people living with dementia and demonstrated the ability of family to differentiate between dementia‐specific symptoms and those of delirium.

A feasibility study of the family‐centred, function‐focused care intervention (Fam‐FFC) actively engaged the patients and family caregiver (FCGs) in care‐related decision making (Boltz et al. 2015). The intervention worked with dyads of hospitalised medical patients with dementia and FCGs (Boltz et al. 2015). The primary hypothesis was that Fam‐FFC would improve Activities of Daily Living (ADL) performance in patients with dementia at 60 days post discharge. It was also hypothesised that exposure to Fam‐FFC would result in less severity of delirium and a greater return to pre‐hospitalisation performance. Initial findings suggested patients on Fam‐FFC units had better ADL and walking performance and less seventy of delirium. Preparedness for caregiving was evaluated, by assessing the quality (mutuality) of the caring relationship, caregiver anxiety and depression. Results identified a significant increase in preparedness for caregiving but no significant effect of the intervention on depression, mutuality or role strain.

## 5 Discussion

The systematic scoping review assessed emerging evidence from 15 studies across five countries that reported on supportive interventions involving family carers with adults with or at risk of DSD in acute hospital settings.

The included studies were predominantly non‐matched, quasi‐experimental interventions. There was a range of intervention approaches that focused on educational resources, individual staff or whole system approaches. Limited studies reported in any detail on family carer involvement with some relying solely on family feedback. Most of the studies were clinically led with limited input from family carers or their representatives in the design.

The review findings demonstrated recognition of the importance of the family or their proxy as a way of supporting staff when providing care to people living with dementia with delirium. Only two studies considered the quality or mutuality of the patient–family–carer relationship or how confident family felt to raise concerns (Boltz et al. 2015; Kang et al. 2017). Establishing how well family members knew the patient, who was best placed to be the key informant and the support family members needed to raise issues, received little attention. Other studies have identified the importance of recognising who knows the patient best and addressing family concerns about causing staff additional work (Abbott et al. 2022; Anantapong et al. 2022; Quinn et al. 2019). A recent review of nurses' experiences of managing delirium (not DSD) in acute care settings found nurses did not prioritise this work even though the evidence supports active prevention and intervention. They argued for organisational support to improve nurse awareness and practice (Lim et al. 2022).

Regardless of the type of intervention, family appreciated having the opportunity to be involved. The studies with educational interventions increased staff knowledge of the patient and preparedness for caring for this sometimes‐complex patient group but how this led to improved outcomes was not demonstrated.

Further work is needed to understand how family involvement is planned and supported and to understand the resources that facilitate their involvement (educational materials and digital resources). A recurring issue in the literature was whether family involvement reduced demands on staff time. However, for family to be adequately supported and incorporated into the working practices of the ward suggests this will require extra staff engagement with the patient and their family. The review reiterated the potential of involving family members in the care of people with DSD. However, there was minimal evidence of involving family in the study design or commenting on what was helpful.

The review findings on the benefits of involving family in acute care settings complement work on patient–carer dyads and relationship‐centred working for older adults with dementia and their family carers in other settings reported by (Birkenhäger‐Gillesse et al. 2020, 2022). Both have shown the value of thinking about the patient–carer dyad and the need for further research to establish what kind of interventions are effective. It also raises further questions about staff knowledge and understanding of DSD and if this needs to be addressed first before involving family members (Soun et al. 2023).

The review included papers (Ayton et al. 2020; Bateman et al. 2016; Blair et al. 2018; Wong Shee et al. 2014) that had focused on volunteers working with staff in lieu of family carer support. This is perhaps not equivalent as the papers demonstrated that the volunteers became an extension of the nursing team rather than providing the individualised care and knowledge or the patient that comes from being a family carer. They did, however, demonstrate what was possible with additional training and support and the kind of work that would be needed to ensure good outcomes for all involved.

## 6 Limitations

The literature search was limited to articles published in English, creating a possible language bias. It is possible that interventions were missed that included family members that did not make this explicit in the title or abstract. The search strategy was limited by publication date, including literature between 2009 and2019, extended to include 2019–2023. These limitations may have potentially excluded papers with findings related to the study aim. The inclusion criteria did not require a definition of old age—future work should consider a cut‐off age for how older adults were defined.

## 7 Conclusion

There is a consensus that family members or family proxies should be involved in supporting people living with dementia when in hospital, especially when at risk of or with delirium. By focusing on the impact of supportive interventions involving family carers in care delivery, this review has highlighted the need for more research on how to involve family members in the design of interventions and enable nursing staff to work in ways that are see the family member as a partner in care.

## Author Contributions

All authors have accepted responsibility for the entire content of this manuscript and consented to its submission to the journal, have reviewed all the results and have approved the final version of the manuscript.

## Conflicts of Interest

C.A.‐G. is supported by Princess Alexandra Hospital Harlow. C.G. is an NIHR senior investigator. The views expressed are those of the authors and not necessarily those of the NHS, the NIHR or the Department of Health and Social Care.

## Data Availability

The data that support the findings of this study are available from the corresponding author upon reasonable request.

## Appendix 1

### Search strategy

**Table:**

Databases	Interfaces	Results	Date of Search
Cinahl (scoping)	HDAS	785	05.09.2018
PsycINFO (scoping)	HDAS	36	13.12.2018
PubMed (scoping)	PubMed	52	13.12.2018
Medline (scoping)	HDAS	203	09.01.2019
Medline	HDAS	267	17.01.2019
PsycINFO	ProQuest	33	23.02.2023
Cochrane	Cochrane	110	23.02.2023
Cinahl Complete	EBSCO	92	23.02.2023
Medline (1946–2023 February 17)	OVID	116	20.02.2023
Embase (1974–2023 February 17)	OVID	336	20.02.2023

### Deduplication

Number of references: 687

After deduplication in endnote: 471

### Search Terms

**Table:**

Dementia Delirium cognitive impairment* cognitive disorder*	hospital* ward* communit*	nurs*
Dementia [MeSH] Dementia [APA PsycINFO Thesaurus] MH Dementia Dementia [Emtree] Delirium [MeSH] Delirium [APA PsycINFO Thesaurus] MH Delirium Delirium [Emtree] “Cognition Disorders” [MeSH] MH “Cognition Disorders” “Cognition Disorders” [Emtree] “Cognitive Impairment” [APA PsycINFO Thesaurus] “Cognitive Disorders” [APA PsycINFO Thesaurus]	“Hospital Departments/nursing” [MeSH] “Hospitals, District” [MeSH] “Hospitals, District” [Emtree] “Health Facility Environment” [MeSH] MH “Health Facility Environment” “Health Facility Environment” [Emtree] “Community Health Services” [MeSH] “Community Health Services” [Emtree] MH “Community Health Services” Hospitals [APA PsycINFO Thesaurus] MH Hospitals “Hospital Environment” [APA PsycINFO Thesaurus] “Community Services”[APA PsycINFO Thesaurus] MH “Hospital Units”	Nursing [MeSH] Nursing [APA PsycINFO Thesaurus] MH Nurses

### Pubmed

(((((delirium[Title/Abstract]) AND dementia[Title/Abstract])) AND (“Dementia”[Mesh]) AND “Delirium”[Mesh]))) AND ((“hospital departments/nursing”[MeSH Terms]) OR hospital*)

### Medline

((((exp “COGNITION DISORDERS”/ OR (cognitive ADJ3 impairment).ti,ab OR (“cognitive impairment*”).ti,ab OR (“cognitive disorder*”).ti,ab OR (cognitive ADJ3 disorder*).ti,ab OR exp. DEMENTIA/ OR (dementia).ti,ab) AND (exp DELIRIUM/ OR (delirium).ti,ab)) AND (exp “HOSPITALS, DISTRICT”/ OR (hospital*).ti,ab OR exp. “HEALTH FACILITY ENVIRONMENT”/ OR (ward*).ti,ab)) NOT (exp “COMMUNITY HEALTH SERVICES”/ OR (communit*).ti,ab)) AND (exp NURSING/ OR (nurs*).ti,ab)

### PsycINFO

(((((MAINSUBJECT.EXACT.EXPLODE(“Delirium”) OR tiab(delirium)) AND ((MAINSUBJECT.EXACT.EXPLODE(“Cognitive Impairment”) OR (tiab(cognitive NEAR/3 impairment) OR tiab((“cognitive impairment” OR “cognitive impairments”))) OR (tiab(cognitive NEAR/3 disorder*) OR tiab((“cognitive disorder” OR “cognitive disorders”)))) OR (MAINSUBJECT.EXACT.EXPLODE(“Dementia”) OR tiab(dementia)))) AND ((MAINSUBJECT.EXACT.EXPLODE(“Hospitals”) OR tiab(hospital*)) OR (MAINSUBJECT.EXACT.EXPLODE(“Hospital Environment”) OR tiab(ward*)))) NOT (MAINSUBJECT.EXACT(“Community Services”) OR tiab(communit*))) AND (MAINSUBJECT.EXACT.EXPLODE(“Nursing”) OR tiab(nurs*))) AND pd(20190101‐20231231)

### Cochrane

((((Exp all [Cognition Disorders] OR cognitive near/3 impairment OR “cognitive impairment*” OR “cognitive disorder*” OR cognitive near/3 disorder*) OR ((Exp all [Dementia] OR dementia) OR (Exp all [Delirium] OR delirium))) AND (Exp all [Hospitals, District] OR hospital* OR Exp all [Health Facility Environment] OR ward*)) NOT (Exp all [Community Health Services] OR communit*)) AND (Exp all [Nursing] OR nurs*)

Limit: publication date between January 2019 and December 2023

### CINAHL Complete

(((((MH “Cognition Disorders+”) OR (TI cognitive N3 impairment OR AB cognitive N3 impairment) OR (TI “cognitive impairment” OR AB “cognitive impairment”) OR (TI “cognitive disorder*” OR AB “cognitive disorder*”) OR (TI cognitive N3 disorder* OR AB cognitive N3 disorder*)) OR ((MH “Dementia+”) OR (TI dementia OR AB dementia))) AND (MH “Delirium”) OR (TI delirium OR AB delirium) AND ((MH “Hospitals+”) OR (TI hospital* OR AB hospital*) OR ((MH “Health Facility Environment”) OR (MH “Hospital Units+”)) OR (TI ward* OR AB ward*))) NOT ((MH “Community Health Services+”) OR (TI communit* OR AB communit*))) AND ((MH “Nurses+”) OR (TI nurs* OR AB nurs*))

Limit: publication date between January 2019 and December 2023

### Embase

((((exp Cognition Disorders/ OR ((cognitive adj4 impairment).ab,ti. OR “cognitive impairment*”.ab,ti. OR “cognitive disorder*”.ab,ti. OR (cognitive adj4 disorder*).ab,ti.))) OR (exp Dementia/ OR dementia.ab,ti.)) AND (exp Delirium/ OR delirium.ab,ti.)) AND ((exp Hospitals, District/ OR hospital*.ab,ti. OR exp. Health Facility Environment/ OR “ward*”.ab,ti.) NOT (exp community health services/ OR “communit*”.ab,ti.))) AND (exp Nursing/ OR “nurs*”.ab,ti.))

## Appendix 2

**TABLE A1:** Supportive interventions and their outcomes.

Intervention	Intervention	Aim of the intervention	Type of support provided	Intervention provider	Material	Family member	Main findings	Author and publication
Implementation based on						Outcome measurement instrument		
Hospital Elder Life Program (HELP)	Surveys and focus groups and interviews (hospital staff, volunteers, patients and caregivers) were conducted simultaneously.	To explore the acceptability of the The Volunteer Dementia and Delirium Care (VDDC) programme	1‐1 companionship for patients with dementia and/or delirium	Volunteers	N/A	Patient and caregiver dyad exploratory interviews about patient experience, and perspectives of the appropriateness of the VDDC programme	Overall participants believed the VDDC was acceptable and would be of benefit to the patients	Ayton et al. (2020)
Volunteer programme	Person‐centred volunteer programme	To address the challenge's faced by staff in an acute rural hospital when providing person‐centred care for patients with dementia/delirium through a volunteer programme	Training volunteers to provide personal support, then measuring outcomes of the intervention Activity's Making patients comfortable Providing food/drink Talking, assisting with menu selection, orientation	Volunteers	Training programme Resource manual Training materials	Not reported	Possible to implement at a low cost volunteer programme to support quality of care Patient outcomes, reduction in use of antipsychotics, increase in analgesia Length of stay halved	Bateman et al. (2016)
Volunteer programme	Person‐centred volunteer programme	Evaluate the clinical outcomes for patients with dementia, delirium, or at risk of delirium supported be the person‐centred volunteer programme in rural acute hospitals	Volunteers training programme	Volunteers	Training sessions Volunteer dementia and delirium care training resource	Carers rated how helpful having a volunteer was on a Likert scale. 96% (*n* = 77) rated the volunteer intervention as helping ‘a lot’ (89%) or ‘a little’ (8%)	The Dementia and Delirium Care with Volunteers programme was successfully implemented in 7 rural hospitals of varying sizes and resulted in reductions in 28 day readmission and specialling rates. No increases in falls, pressure ulcers, mortality or institutionalisation indicates the intervention is safe. The implementation package and mentoring resulted in consistent implementation and results across all sites.	Blair et al. (2018)
Family‐cantered approach to care	Testing the feasibility and preliminary efficacy of a family‐centred, function‐focused care intervention	To see if hospital acquired disabilities and complications in people with dementia can be reduced through a family‐centred function‐focused care	Implementation of Fam‐FFC, which included (environmental and policy assessment, staff education/training, ongoing motivation/training of staff)	Family‐centred resource nurse Research team Unit champion	Comparative repeated measure study testing the feasibility and preliminary efficacy of the family‐centred, function‐focused care intervention	Patient outcomes—less delirium severity, 2 weeks and 2 months post discharge. Walking performance, length of stay. Better ADL performance Family care–giver outcomes—significant increase in preparedness for care giving. No significant effect of impact on FC depression	The findings from this study support the feasibility of Fam‐FFC and show preliminary efficacy that implementation of Fam‐FFC can improve outcomes for both hospitalised patients with dementia and their FCGs. Specifically patients in Fam‐FCC units had better ADL and walking performance, less severity and duration of delirium and fewer hospital readmissions. FCSs on Fam‐FFC units showed a significant increase in preparedness for caregiving and less anxiety from admission to 2 months post discharge.	Boltz et al. (2015)
Fundamentals of care	High‐quality fundamentals of care	To clarify how high‐quality fundamentals of care for people with dementia and/or delirium were practised in a specialist older people evaluation and management unit	Staff Family carers Researchers	*N* = 3 patients *N* = 5 family members *N* = 2 managerial staff *N* = 35 Clinical staff *N* = 5 non‐clinical staff members	N/A	Family valued fundamentals of care	High‐quality fundamental care for people with dementia and/or delirium in hospital and their families was associated with the special space of the hospital unit; an aptitude for people with dementia; a capacity to translate person‐centredfundamentals of care from rhetoric to reality; and an appreciation for teamwork.	Collier et al. (2020)
Education programme	A computerised support component of delirium screening as part of an intervention strategy	Test the feasibility of the computerised delirium support screens.	Education programme Computerised decision support Unit champion Feedback mechanism	*N* = 15 Patients/family care givers	Computerised decision support component intervention strategy	Satisfaction survey family members *N* = 13, 6 felt patient strongly benefitted from intervention, 2 did not, 5 were uncertain	MMSE scores improved or stayed the same from admission to discharge Only half the nurses attended he education module, nurses indicated they need more education in delirium	Fick et al. (2011)
Approach to care delivery	Participants were randomised to a specialist medical and mental health unit, designed to deliver best practice care for people with delirium or dementia, or to standard care (acute older people or general medical wards)	To compare the care in specialist medical and mental health unit compared with standard care for older people with cognitive impairment admitted to general hospital	Joint staffing by medical and mental health professionals Enhanced staff training in delirium, dementia and person‐centre care in dementia Provision of organised purposeful activity Environmental modification Proactive and inclusive approach to carers	2 consultant geriatricians Specialist mental health staff Physiotherapist 3 activities co‐coordinators Speech and language therapist Visiting psychiatrists	Set up a specific ward for the research	Carers satisfaction (measured on 10 dimensions of care) Number of days spent at home(or in care home) after randomisation Patients mood and engagement on the ward measured by direct observation	Assumption made there would be improvements in length of stay, well‐being, communication, better recognition of mental health, delirium would reduce length of stay/readmission, but the results were modest and could have occurred by chance Carers satisfaction identified through telephone calls Family carers randomised to specialist unit were significantly more satisfied with overall care, nutrition, dignity and respect and discharge arrangements Tail of severe dissatisfaction in both groups, twice as frequent in standard care 0.004	Goldberg et al. (2013)
Educational approach	Education programme	To identify the effect of an educational programme on Registered Nurses efforts to involve family caregivers in delirium care	Education Brochure Advice re activities	RN'S Family caregivers	Educational delirium brochure Family involvement	Most family members reported a positive experience. Some felt an over reliance on caregivers	Most RN's reported a positive experience in involving RN initiated interactions in delirium care, three factors affected their involvement. 1, Delirium brochure 2, Sense of responsibility 3, Expectations of RNS. Barriers—lack of time and over reliance on family caregivers	Kang et al. (2017)
Family carer approach to delirium assessment	Family carers programme	To test the feasibility of Fam‐FFC and examine its impact among persons with and without dementia and their FCGs in the ED To examine the ability of the family‐rated Family‐Confusion Assessment method (FAM‐CAM) to identify delirium in the emergency department (ED) among patients with and without dementia, as compared to the reference‐standard Confusion Assessment Method (CAM)	Caregivers provided informed consent Use of FAM‐FFC tool Family carer interviews	Patients with and without dementia Family carers	FAM‐CAM Tool	Not reported	The FAM‐CAM had moderate sensitivity (57%; 95% confidence interval [CI]: 39%–74%) but higher specificity (83%; 95% CI: 75%–92%), when compared to the reference standard CAM. Among patients with dementia, the FAM‐CAM showed higher sensitivity (with dementia, 61%; 95% CI: 41%–81% vs. without dementia, 43%; 95% CI: 6%–80%) and lower specificity (with dementia, 74%; 95% CI: 60%–89% vs. without dementia, 91%; 95% CI: 82%–99%)	Mailhot et al. (2020)
Person‐centred care	Multicomponent intervention programme	To assess the efficacy of multicomponent intervention in delirium prevention	Education Provision of a clock Avoidance of sensory impairment Presence of familiar objects Reorientation Extended visiting rights	Family	Information technology Educational modules	6 family members felt the patient strongly benefited from the intervention	Primary outcomes—presence of delirium assessed by CAM Secondary outcome—incidence of falls during hospital stay and complications derived from them	Martinez et al. (2012)
Education	Family‐focused delirium education initiative	To test the impact of the development of a delirium brochure to provide delirium education to family caregivers and empower them to be an integral part of the healthcare system	Charge nurse, nurse educator and the early nurse detection of DSD study staff provided frontline nurses information about the brochure	Expert panel consisted of 2 medical doctors, 4 doctorial nursing students and 4 ANP Patient and carer feedback not assessed during iterative process	Development of a brochure	Lack of evaluation of family caregivers. Analysis of the survey showed a drive to use brochure but lacked current dissemination of the brochure by nursing staff	Analysis showed drive to use the brochure but lack of dissemination by nursing staff 86% staff would recommend the brochure to other nurses	Paulson et al. (2016)
Education	Implementation of an innovative interprofessional learning programme	To test the impact of a 2‐day programme ‘Learning about the patient’	Learning directly from patients and carers. Patients taught sections of the course, production of two patient videos (dementia/delirium patient experience). Interprofessional teaching and a clear focus on action learning	Patients Carers Nursing staff	Video production	Practice change not evaluated Carers involved in developing the teaching but outcomes not evaluated	Significant improvement in the learners confidence in managing issues relevant to this patient group (*p* < 0.001) Positive changes in knowledge and attitudes, as suggested by the posters	Teodorczuk et al. (2014)
Simulated family presence	Pre‐recorded video messages from family's for patients with dementia and delirium	To examine the effect of simulated family presence through pre‐recorded video messages on the agitation level of hospitalised, delirious, acutely agitated patients	Development of a family video message	Patient and family	Video	No family outcomes/analysis reported	Both family video and nature groups –significant change in median agitation scores over the 4 time periods (*p* < 0.001) Family video group lower median agitation scores during intervention period (*p* < 0.001) Greater proportion, 94%, experiencing reduction in agitation from pre‐intervention to during intervention, than those viewing nature video 70%, usual care 30%	Waszynski et al. (2018)
Volunteer programme	Volunteer diversional therapy programme	To evaluate the feasibility and acceptability of a volunteer diversional therapy programme for patients with cognitive impairment undergoing inpatient rehabilitation	A diversional therapy assessment for each participant Volunteers provided orientation and diversional therapy activities, using appropriate communication for interaction with a person with memory or thinking difficulties	Volunteers	Personal Profile for each patient was collected	The patient and carer participants reported that the programme provided opportunities for social activity and interactions.	There was high acceptability of the volunteer programme by the nursing staff, patients and their carers and the volunteers, all of whom perceived positive patient outcomes. However, there were also a number of recommended improvements related to staff engagement with the programme and the volunteers' education and training.	Wong Shee et al. (2014)
Education	Early nurse detection of Delirium Superimposed on Dementia (END_DSD)	Sub‐analysis of data collected from a 5‐year, cluster‐randomised trial of Early Nurse Detection of Delirium Superimposed on Dementia (END‐DSD)	(1) Nursing education; (2) computerised decision support embedded within the electronic health record (EHR); (3) a designated unit delirium champion; and (4) weekly rounding sessions led by an advanced practice nurse	Advanced practice nurse	Education Delirium Rounding forms	Cannot determine if there is a relationship between provision of PCC and delirium occurrence. Family carers not evaluated	Significant improvement in the learners confidence in managing issues relevant to this patient group (*p* < 0.001) Positive changes in knowledge and attitudes, as suggested by the posters	Yevchak et al. (2017)

## Appendix 3

**TABLE A2:** Joanna Briggs Institute critical appraisal tool for qualitative research (Lockwood et al. 2015).

Questions Is there congruity between the stated philosophical perspective and the research methodology? Is there congruity between the research methodology and the research questions or objectives? Is there congruity between the research methodology and the methods used to collect data? Is there congruity between the research methodology and the representation and analysis of the data? Is there congruity between the research methodology and the interpretation of the results? Is there a statement locating the researcher culturally or theoretically? Is the influence of the researcher on the research, and vice versa addressed? Are participants, and their voices, adequately represented? Is the research ethical according to current criteria or, for recent studies, and is there evidence of ethical approval by an appropriate body? Do the conclusions draw on the research report flow from the analysis, or interpretation, of the data?

Citation	Study design	JBI critical appraisal questions													Overall
		Q1	Q2	Q3	Q4	Q5	Q6	Q7	Q8	Q9	Q10	Q11	Q12	Q13	
Ayton et al. (2020)	Mixed method	Y	Y	Y	Y	Y	N	N	Y	Y	Y	N/A	N/A	N/A	Include
Bateman et al. (2016)	Quasi‐experimental	Y	Y	Y	N	N	Y	Y	Y	Y	U	N/A	N/A	N/A	Include
Blair et al. (2018)	Mixed methods	Y	Y	Y	N	N	Y	Y	Y	Y	Y	N/A	N/A	N/A	Include
Boltz et al. (2015)	Comparative repeated measures study	Y	Y	Y	N	N	N	N	U	Y	Y	N/A	N/A	N/A	Include
Collier et al. (2020)	Qualitative	Y	Y	Y	Y	Y	Y	N	Y	Y	Y	N/A	N/A	N/A	Include
Fick et al. (2011)	Prospective cohort	Y	Y	Y	N	N	Y	N	Y	U	Y	N/A	N/A	N/A	Include
Kang et al. (2017)	Mixed methods	N	Y	Y	Y	N	N	U	U	Y	Y	N/A	N/A	N/A	Include
Mailhot et al. (2020)	Validation	Y	Y	Y	Y	U	N	N	Y	U	Y	N/A	N/A	N/A	Include
Paulson et al. (2016)	Qualitative	Y	Y	U	U	Y	N	N	Y	Y	Y	N/A	N/A	N/A	Include
Teodorczuk et al. (2014)	Grounded theory	Y	Y	Y	Y	Y	N	N	N	Y	Y	N/A	N/A	N/A	Include
Wong Shee et al. (2014)	Qualitative	Y	Y	Y	U	U	N	N	Y	N	Y	N/A	N/A	N/A	Include
Yevchak et al. (2017)	Exploratory descriptive	Y	Y	Y	U	Y	N	N	U	Y	Y	N/A	N/A	N/A	Include

*Note:* N, no; N/A, not applicable; U, unclear; Y, yes.

**TABLE A3:** Joanna Briggs Institute critical appraisal tool for RCT (Tufanaru et al. 2020).

Questions Was true randomisation used for assignment of participants in treatment groups? Was allocation to treatment groups concealed? Were treatment groups similar at the baseline? Were participants blind to treatment alignment? Were those delivering treatment blind to treatment assignment? Were outcomes assessors blind to treatment alignment? Were treatment groups treated identically rather than the intervention of interest? Was follow‐up complete and if not, were differences between groups in terms of their follow‐up adequately described and analysed? Were participants analysed in the groups to which they were randomised? Were outcomes measured in the same way for treatment groups? Were outcomes measured in a reliable way? Was appropriate statistical analysis used? Was the trial design appropriate, and any deviations from the standard RCT design (individual randomisation, parallel groups accounted for in the conduct and analysis of the trial?)

Citation	Study design	JBI critical appraisal questions													Overall
		Q1	Q2	Q3	Q4	Q5	Q6	Q7	Q8	Q9	Q10	Q11	Q12	Q13	
Goldberg et al. (2013)	RCT	N	Y	Y	Y	N	U	N	Y	Y	Y	Y	Y	Y	Include
Martinez et al. (2012)	RCT	Y	Y	Y	Y	Y	Y	N	Y	Y	N	Y	N	Y	Include
Waszynski et al. (2018)	RCT	Y	Y	Y	N/A	Y	Y	Y	N	Y	Y	Y	Y	Y	Include

*Note:* N, no; N/A, not applicable; U, unclear; Y, yes.
